# Cuticular surface damage of *Ascaridia galli* adult worms treated with *Veitchia merrillii* betel nuts extract *in vitro*

**DOI:** 10.14202/vetworld.2017.732-737

**Published:** 2017-07-03

**Authors:** Ummu Balqis, Muhammad Hambal, Rinidar, Farida Athaillah, Ismail, Azhar, Henni Vanda, Darmawi

**Affiliations:** 1 Laboratory of Pathology, Faculty of Veterinary Medicine, Syiah Kuala University, Banda Aceh, Aceh, Indonesia; 2 Laboratory of Parasitology, Faculty of Veterinary Medicine, Syiah Kuala University, Banda Aceh, Aceh, Indonesia; 3 Laboratory of Pharmacology, Faculty of Veterinary Medicine, Syiah Kuala University, Banda Aceh, Aceh, Indonesia; 4 Laboratory of Veterinary Public Health, Faculty of Veterinary Medicine, Syiah Kuala University, Banda Aceh, Aceh, Indonesia; 5 Laboratory of Physiology, Faculty of Veterinary Medicine, Syiah Kuala University, Banda Aceh, Aceh, Indonesia; 6 Laboratory of Research, Faculty of Veterinary Medicine, Syiah Kuala University, Banda Aceh, Aceh, Indonesia; 7 Laboratory of Microbiology, Faculty of Veterinary Medicine, Syiah Kuala University, Banda Aceh, Aceh, Indonesia

**Keywords:** anthelmintics, *Ascaridia galli*, cuticle, *Veitchia merrillii*

## Abstract

**Aim:**

The objective of this research was to *in vitro* evaluate the cuticular surface damage of *Ascaridia galli* adult worms treated with ethanolic extract of betel nuts *Veitchia merrillii*.

**Materials and Methods:**

Phytochemical screening was done using FeCl_3_, Wagner and Dragendorff reagents, NaOH, MgHCl, and Liebermann–Burchard reaction test. Amount of 16 worms were segregated into four groups with three replicates. Four worms of each group submerged into phosphate buffered saline, 25 mg/ml, and 75 mg/ml crude ethanolic extract of *V. merrillii*, and 15 mg/ml albendazole. The effect of these extract was observed 40 h after incubation as soon as worms death. The worms were sectioned transversally and were explored for any cuticular histopathological changes in their body surface under microscope.

**Results:**

We found that the ethanolic extract of *V. merrillii* betel nuts contains tannins, alkaloids, flavonoids, triterpenoids, and saponins. The ethanolic extract of betel nuts *V. merrillii* induces surface alterations caused cuticular damage of *A. galli* adult worms.

**Conclusion:**

We concluded that ethanolic extract of betel nuts *V. merrillii* possess anthelmintic activity caused cuticular damage of *A. galli* adult worms.

## Introduction

Infestation by intestinal roundworm *Ascaridia galli* called ascaridiosis presents a main threat to the indigenous poultry production in most parts of the world. Ascaridiosis is prevalent in several countries such as Egypt [1], Bangladesh [2,3], Germany [4], Jordan [5], India [6,7], Iraq [8], and Indonesia [9,10]. Heavily infected chickens may cause damaging the integrity of intestinal villi [7,8], intestinal mucosal defense can be changed [10], lumen contained thick white pasty mucous, enteritis, and intestinal wall appeared to be thickened with mucosa giving a velvety appearance [7], and nutrient utilization can be show affected which decreased weight gain [11]. *A. galli* also affects millions of poultry resulting in considerable economic losses in domestic and farmyard bird. A number of anthelmintic compounds have been used in controlling the parasite infected in animals.

Treatments using commercial anthelmintic drugs are not only expensive but also disadvantages such as the risk of environmental pollution, affect host health, and leads to widespread development of resistance to most of the current anthelmintics [12], for example, the existence of multiple anthelmintic resistance in gastrointestinal nematodes of sheep in Colombia [13]. Anthelmintic resistance is almost cosmopolitan in distribution in almost all species of domestic animals and even in some parasites of human beings [14]. In Punjab (Pakistan), several gastrointestinal parasites, namely, *Haemonchus contortus*, *Trichostrongylus colubriformis, Cooperia curticei, Teladorsagia circumcincta*, and *Oesophagostomum* spp. had resistance on oxfendazole in beetal goats [15]. The control of gastrointestinal nematodes using anthelmintic in cattle leads to the development of *Cooperia punctata*, *Cooperia pectinata*, *Oesophagostomum radiatum*, and *Trichuris* spp. population resistant to moxidectin [16]. The previous experiment reported that in some farms in the northwestern region of São Paulo State, Brazil; there are indications about the resistant of *Cooperia* spp. and *Haemonchus* spp., especially to ivermectin. The resistance to albendazole and levamisole was also observed in cattle [17]. Regarding the prevalence of anthelmintic resistance in gastrointestinal nematodes, Demeler *et al*. [18] suggested that testing of anthelmintic efficacy should be performed more intensively due to the possible insufficient efficacy of ivermectin and benzimidazole on cattle farms in Germany, Belgium and Sweden.

Because of the problems arising from the use of conventional anthelmintics highlighted above, some investigators have mentioned the importance to find alternative ethnomedicinal extracted from plant materials. Many plants species used worldwide in traditional medicine as better alternative is gaining significance. In the study of El-Sherbini and Osman [19] evaluated aqueous extracts of immature fruits of the mango *Mangifera indica* L. for inhibition of larval development indicate that this fruit could assist *Strongyloides stercoralis* control. For anthelmintic properties, *in vitro* and *in vivo* employed by Hussain *et al*. [20] showed that both *Trianthema portulacastrum* and *Musa paradisiaca* possess strong anthelmintic activity against *Oesophagostomum columbianum*, *Trichuris ovis*, *Trichostrongylus* spp., and *H. contortus*. In another study using plant *Lippia sidoides*; CamurÇa-Vasconcelos *et al*. [21] noticed that the efficacy of the *L. sidoides* essential oil on sheep gastrointestinal nematodes, *H. contortus*. The traditional system of treating parasitic diseases hold a great promise as a source of easily available effective anthelmintic agents to the people particularly in tropical developing countries, including Indonesia.

Because of easy availability, *A. galli* adult worms are used as suitable models for screening of anthelmintic drug. Various researchers observed *in vitro* anthelmintic effects of Bishkatali *Azadirachta indica* (neem) leaves extract against *A. galli* [2,3,22,23]. Regarding anthelmintic activity against *A. galli*, Ali *et al*. [24] reported that the parasiticidal effect of albendazole was less in comparison with saponins extracted from *Teucrium stocksianum*. Abdelqader *et al*. [25] reported that citrus peels extracts have potential anthelmintic properties against *A. galli*. Alrubaie [8] used the *Curcuma longa* L. roots alcoholic extract against worm *A. galli*. Ahmad *et al*. [6] and Salam [7] demonstrated that the effect of *Mentha longifolia* leaf extract might contribute to the development of effective against *A. galli*. Previously, Islam *et al*. [22] suggested that dust of Bishkatali (*Polygonum hydropiper*) leaves can be used with litter for inhibition of development of *A. galli* eggs and fresh juice and extract of *A. indica* and papaya (*Carica papaya*) may be impregnated in litter and used after sundry.

*Veitchia merrillii* betel nuts from the *Palmaceae* family is a tropical fruit, which is also called “Christmas Palm” because its fruits become bright scarlet and tend to be that color in winter and is widely distributed in different parts of the world. The fatty acid composition, determined by gas chromatography, rendered that palmitic, oleic, and linoleic acids were the major compounds in extracts from the *V. merrillii* species [26]. Our previously study indicated that *V. merrillii* betel nuts extract reduced motility, paralysis, and death time *A. galli* adult worms *in vitro*, is possible to be potential for developing herbal-based anthelmintic to control *A. galli* [27,28]. But unfortunately, the research did not show any histological alterations in their cuticle architecture after exposure to *V. merrillii* extract. It is hoped that this study may improve knowledge of the anthelmintic activity of *V. merrillii* nuts extract caused any histological alterations in the tissue of *A. galli* adult worms using light microscopy.

## Materials and Methods

### Ethical approval

This research was approved by the Animal Ethics Committee of Faculty of Veterinary Medicine, Syiah Kuala University (Approval No. 10/KEPH-C/III/2016). The chickens from which *A. galli* adult worms were collected, were handled in accordance with good animal practices required by the Animal Ethics Committee and the guidelines of local rules and regulations.

### Extraction of V. merrillii betel nuts

*V. merrillii* betel nuts were procured from around yard areas of Banda Aceh. The plant was identified in the Herbarium, Department of Biological Sciences, Syiah Kuala University, and voucher specimen number of 63. The betel nuts were pulverized into powder by pounding in a mortal using a pastel. The powder of betel nuts (250 g) was extracted using ethanol as explained by Hussain *et al*. [20] and Sarojini *et al*. [29]. Dry powdered betel nuts material (1.5 kg) was soaked in 6 L of 70% aqueous (aq.) ethanol by cold maceration at room temperature for 3 days. The filtrate collected through muslin cloth by repeated soaking of dry powder of betel nuts materials. The extract was then concentrated by evaporation under a temperature 40°C to dryness as explained by Hussain *et al*. [20] and Anthikat *et al*. [30] with certain modification. The extract was then resuspended in distilled water and diluted to the desired concentration.

### Phytochemical screening

Phytochemical screening was done for verifying tannins, alkaloids, flavonoids, saponins, terpenoids, and steroids groups present in the extract. Tannins were tested using FeCl_3_. Alkaloids were verified using Wagner and Dragendorff reagents. Flavonoids and saponins were determined by the NaOH, concentrated sulfate acid, and MgHCl tests. Terpenoids and steroids were verified by the Liebermann–Burchard reaction as described by El-Sherbini and Osman [19], Balqis *et al*. [27], Hamzah *et al*. [28], Sarojini *et al*. [29], Suleiman *et al*. [31].

### Collection of A. galli roundworms

The *A. galli* roundworms acquired from intestines of native breed domestic fowl (*Gallus domesticus*) which were procured from the local chickens in Banda Aceh. The collected intestines immediately were taken to the Research Laboratory of the Faculty of Veterinary Medicine of Syiah Kuala University. All of the intestines were dissected longitudinally and submerged in normal saline solution. All worms visible to the naked eye were collected using a pair of forceps. *A. galli* adult worms approximately of having an average size as 5-7 cm were collected in and thoroughly washed with 9% neutral phosphate-buffered saline. The most robust specimens of the lot were chosen for *in vitro* anthelmintic activity testing as described by Balqis *et al*. [27], Hamzah *et al*. [28], Lalchhandama [32].

### Anthelmintic activity

The effect of the *V. merrillii* betel nuts extract was assayed on the nematode. Amount of four *A. galli* adult worms placed in Petri dishes. The worms were maintained in normal saline solution. The prelabeled extracted from *V. merrillii* betel nuts were tested at concentration of 25 and 75 mg/ml. We used a positive control 25 ml albendazole (standard drug) at concentration of 15 mg/ml. Meanwhile, for negative control, we used 25 ml distilled water. All plates were incubated in a room temperature for 48-h [24,27,28].

### Tissue preparation for histological alterations protocol

Each roundworm procured from the Petri dishes of each group were set for paraffin embedding and were dissected for histological sections through the middle portion of the body. This process was performed for each roundworm using sterile instruments for each dissection. The middle’s segment was fixed in 10% buffered normal formalin. Fixed samples were dehydrated in the ascending concentrations of ethanol (50%, 60%, 70%, 80%, 96%, and 100%). The samples were cleared in xylol and were embedded in paraffin wax. The worms were sectioned transversally using a microtome. The sections were deparaffinized with three changes of xylene (I, II, and III) for 3 min of each and were explored for any histopathological changes in their body surface under microscope as described by Darmawi *et al*. [9].

Three of each histological sections (3-5 µm of thickness) were stained with hematoxylin and eosin (pH 0.1) (Sigma). After washing, sections were counterstained with eosin and mounted with Entellan^®^ as described by Darmawi *et al*. [9] and Darmawi *et al*. [10]. The histological alterations in the tissue of *A. galli* were investigated on each section under light microscopy (Olympus, Tokyo - Japan) using an eyepiece square graticule (eyepiece 10×, objective 10×), and data expressed as abnormalities and photographed as described by Jeyathilakan *et al*. [33], Jiraungkoorskul *et al*. [34], Li *et al*. [35], Königová *et al*. [36].

## Results

**Figure-1:**
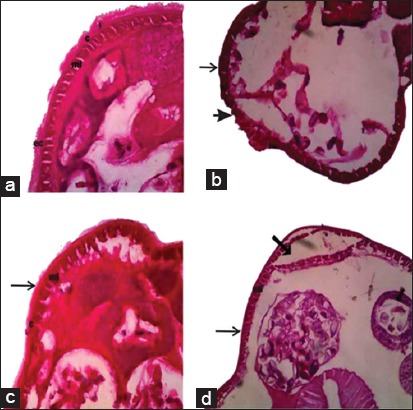
Photomicrograph of sections of Ascaridia galli adult worms (H and E, 10×). (a) Phosphate buffered saline: Appearance of epicuticle, cuticle, and muscle layer, (b) albendazole 15 mg/ml: Depletion epicuticle along with cuticle (thin arrow), damaged cuticle and muscle layer arrowhead), (c) ethanolic extract of Veitchia merrillii betel nuts 25 mg/ml: Depletion epicuticle along with cuticle (thin arrow), (d) ethanolic extract of betel nuts V. merrillii 75 mg/ml: Depletion epicuticle along with cuticle (thin arrow) and irregular shaped cuticle with disruption (thick arrow). ec=Epicuticle, c=Cuticle, ml=Muscle layer.

The fraction extract from dried of *V. merrillii* betel nuts was brown greenish, semisolids with characteristic odor. The extract of *V. merrillii* betel nuts contained tannins, alkaloids, flavonoids, triterpenoids, and saponins but no steroids. Our results showed that the *A. galli* adult worms maintained in normal saline solution had a normal body wall formed by an epicuticle, cuticle, and muscle cells. We found that dramatic change in the form of disorganization and destruction of the epicuticle and cuticle of *A. galli* treated with albendazole. We described that morphological changes of cuticle were observed in most of treated with *V. merrillii* betel nuts extract groups. Comparatively, the concentration of *V. merrillii* betel nuts extract 25 ml/mg showed less effect on cuticle than 75 mg/ml. On the concentration of *V. merrillii* betel nuts extract 25 ml/mg we found the depletion epicuticle along with cuticle. Treatment of *A. galli* worms with *V. merrillii* betel nuts extracts 75 mg/ml resulted in dramatic changes in irregular shaped cuticle with disruption as shown in Figure-1.

## Discussion

The results of this study of the fraction extract from dried of *V. merrillii* betel nuts reflect that there is no difference between our finding and previously study [26]. Tannins, saponins, alkaloids, and flavonoids of ethanolic extracts from *Anogeissus leiocarpus, Khaya senegalensis, Euphorbia hirta* and aqueous extracts from *Annona senegalensis* and *Parquetina nigrescens* according to Ndjonka *et al*. [37] affected *in vitro* growth and survival of nematode parasite, *Caenorhabditis elegans* and filarial from cattle, and *Onchocerca ochengi*. Indeed, tannin and flavonoid compounds extracted from unripe mango, *Mangifera indica* L., were able to inhibit the *in vitro* development of *S. stercoralis* larvae, the endemic intestinal nematode in human [19].

Our finding is a similar to those preliminary phytochemical screening observed by Balqis *et al*. [27] Hamzah *et al*. [28]. However, the mechanisms of action of these extract were not clearly understood. The study of Hassanain *et al*. [1] analyzed the similarity component of *A. galli* body surface. According to some investigators who observed ultrastructurally the body wall of normal *A. galli* adult worm consists of longitudinal muscle layer, syncytial hypodermis, cuticle, and epicuticle [32,38]. In confirmation with our early study regarding somatic of *A. galli*, we found that the cuticle contained material antigenic substances that recognized by immunoglobulin yolk antibody [9]. Previously, Oliveira-Menezes *et al*. [39] shown that the cuticular surface consists of transversal striations along the body of parasite filariasis in human, *Wuchereria bancrofti*.

In the parasitology literature, it is well known that the nematodes cuticle plays an important multi-functional role in perform protective and selective absorption function. Importantly, the cuticle of nematode becomes a primary target site of anthelmintic drugs [40]. In this study, we agree with and support those of Lalchhandama *et al*. [41] who found that *A. galli* adult worms treated with albendazole experiencing destruction of cuticle. A similar outcome was observed the effect anthelmintics showing transversal striations along the body in cuticular of *W. bancrofti* [39]. Previously, Robinson *et al*. [42] explained that albendazole had an effect on blockage of microtubules β-tubulin polymerization causes structural and functional damage in the parasite.

*In vitro* tests using free-living stages of parasitic helminths have been used to evaluate the anthelmintic activity of new plant compounds. Suleiman *et al*. [31] described that methanol extract of *Cassia* *occidentalis* and *Guiera senegalensis* was both positive for tannins, saponins, flavonoids, steroids, and triterpenes. Sarojini *et al*. [29] explained that tannins, alkaloids, flavonoids, triterpenoids, saponins, and steroids screened from *Saraca indica* extract displayed anthelmintic property in a dose-dependent manner against *Pheretima posthuma*. Ali *et al*. [24] found that the crude saponins prepared from *T. stocksianum* have cytotoxic and viability effect of the extract on different helminth species, namely, *A. galli* nematode, *Raillietina spiralis* cestode, and also *P. phostuma* earthworm annelida. The data presented here argue that the chemical compounds screened from *V. merrillii* betel nuts extract are also possible involved in any alterations in the cuticle architecture of the *A. galli*.

A characteristic surface morphology of worm alterations occurs during exposure to medicinal ethnoveterinary plant extract. This hypothesis supported by many previously reports exist about anthelmintics activity of plant extract against helminths. Alterations in the tegumental architecture of parasite *Raillietina echinobothrida*, the cestode of domestic fowl reported by Dasgupta *et al*. [43] who investigated the effect of vermicidal activity of the *Acacia oxyphylla* (leguminosa) stem bark-derived. The typical apoptosis resulting in cellular destruction observed in parasite *R. echinobothrida* exposed to three plant extract: *Potentilla fulgens*, *Alpinia nigra*, and *Millettia pachycarpa* [44]. Jeyathilakan *et al*. [33] reported that the trematode liver fluke *Fasciola gigantica*, a common parasite of the hepatic and bile ducts of herbivore mammals, i.e., buffalo, cattle, sheep, and goats showed blebbing of morphological changes in the tegument layer and breakage of spines after *in vitro* treated with essential oils of *Cymbopogon nardus* (citronella) or *A. indica*.

In respect of our results, the finding is a similar to those observed by Roy *et al*. [38], so it seems the effect of the *A. oxyphylla* extract induced cuticular aberrations in the regular striations and damaged epicuticle along with cuticle in *A. galli* adult worms. These findings are not different from those reported the effect of *Calendula micrantha* extract showing wrinkled surface with loss of striations along the body in cuticular of *A. galli* [1]. The effect of the active compound isolated from *A. oxyphylla* on the nematodes became disorganization of *A. galli* body surface [38]. In addition, the *in vitro* anthelmintic activity of the aqueous and hydroalcoholic extracts of *Mentha longifolia* against *A. galli* could be a potential alternative for treating cases of worms infections in chickens [6].

The mechanism of phytochemical constituents action to combat parasite may exhibit different modes. Lorent *et al*. [45] described that cytotoxic activity of saponins was able to form pores in the cell membrane may disrupt the ionic balance of the cell resulting to the cell lysis and death. Recently, the mechanism of action alkaloid extracted from *Combretum zeyheri* described by Nyambuya *et al*. [46] may be due to inhibition of transport across the cell membranes. In general, the mechanism of anthelmintics activity of plant-based extract might act by interference and combining with the cell membranes to elicit changes in cell composition. This induces membrane destabilization, change of membrane permeability, and loses of membrane potential causing the lysis of cells, which has as a consequence of damaging the cuticle. The presence of phytochemical constituents including tannins, alkaloids, flavonoids, triterpenoids, and saponins extracted from *V. merrillii* betel nuts, potentiating the anthelmintic effects in cell membranes disruption leads to cuticular damage of *A. galli* adult worms.

## Conclusion

It is concluded that the *in vitro* experiments clearly indicate that the *V. merrillii* betel nuts extract contained tannins, alkaloids, flavonoids, triterpenoids, and saponins caused cuticular surface damage of *A. galli* adult worms.

## Authors’ Contributions

D and R conceptualized and designed this research. The research was carried by UB, MH, and FA. The manuscript was written by D and UB. HV, A, and I finalized the manuscript. All authors read and approved the final manuscript.
